# Comparison of Visceral Fat Accumulation and Metabolome Markers among Cats of Varying BCS and Novel Classification of Feline Obesity and Metabolic Syndrome

**DOI:** 10.3389/fvets.2017.00017

**Published:** 2017-02-14

**Authors:** Yuki Okada, Motoo Kobayashi, Masaki Sawamura, Toshiro Arai

**Affiliations:** ^1^Department of Veterinary Bioscience, School of Veterinary Medicine, Nippon Veterinary and Life Science University, Musashino, Japan; ^2^Seijyo Kobayashi Veterinary Clinic, Tokyo, Japan; ^3^Sawamura Veterinary Hospital, Chiba, Japan

**Keywords:** feline obesity, metabolic syndrome, adiponectin, visceral adipose tissue, tumor necrosis factor α, lipotoxicity, obesity classification

## Abstract

As in humans, obesity and its associated diseases represent the most significant threat to the health of veterinary populations. Previous human studies have provided insights into the risk factors of obesity, complex pathogenesis of obesity-associated diseases, and their life-threatening consequences. In humans, the “metabolic syndrome” represents a cluster of metabolic risk factors associated with the development of cardiovascular disease. Risk factors for metabolic syndrome, such as diabetes, obesity, high blood pressure, and its complications increase health-care utilization and medical expenses. Early diagnosis and intervention through preemptive approach is in need, and the new International Diabetes Federation definition of MS serves as the universally accepted diagnostic tool that is accessible in clinical settings. In veterinary populations, especially in cats, similar pathophysiological path and disease progression to the development of MS, such as adipokine dysregulations, chronic inflammation, lipotoxicity, are expected. The aim of this manuscript is twofold. First of all, it presents our preliminary feline obesity study that serves as the basis for discussion of obesity and its metabolic impact on feline population. In this study, we observed the effects of weight gain on energy metabolism using metabolome markers, such as adiponectin (ADN) and proinflammatory cytokines, in correlation with other common biochemical parameters in 14 clinically healthy cats of varying weight status. Further, we evaluated the visceral fat accumulation in three subjects of varying Body Condition Scores *via* computed tomography imaging and laparoscopic examination, and assessed the adipocyte type and size histologically. Mutually antagonizing changes in ADN and visceral adipose tissue (VAT) reflected the pathophysiological derangements leading to MS earlier than the common biochemical predictors such as glucose, liver values, and lipid markers. Second, it proposes the novel diagnostic and classification method of feline obesity and MS, based on the established diagnostic criteria of human MS and the presented study results. The results supported our novel “classification of feline obesity” and “Feline MS diagnostic criteria,” suggesting the need to complement ADN measurement with VAT volume to better understand the pathogenesis of metabolic disturbances in the feline population.

## Introduction

The prevalence of obesity and its associated metabolic diseases has been a growing concern and has become the global epidemic in veterinary medicine (1–4). Obesity is an excessive accumulation of triglyceride (TG) in adipose tissues, due to an energy imbalance where energy intake exceeds energy expenditure. Adipose tissue is not only a mere inert fuel storage but also an important secretory and endocrine organ that actively releases substances, “adiopokines.” Adipokines are involved in a wide array of physiological processes including glucose (GLU) and lipid homeostasis, blood pressure, body weight regulations, and immune functions (5, 6). The dysregulated synthesis of “harmful” adipokines, such as tumor necrosis factor α (TNFα) (7), interleukin-6, and of “beneficial” adipokines, such as adiponectin (ADN) (8) and leptin (9), is involved in the development of obesity-associated syndrome. Obesity-associated diseases include diabetes mellitus (DM), insulin (INS) resistance, lipid profile abnormalities, orthopedic diseases, cardiorespiratory diseases, neoplasia, and shortened life span (10, 11). Even before obesity-associated diseases are clinically apparent, evidences of oxidative stress and low-grade chronic inflammation are present due to increased lipid peroxidation and proinflammatory adipokine secretion from growing adipose tissues. Increased oxidative stress affects adipokine production, and dysregulated production of adipokines plays a crucial role in the development of obesity-associated diseases (11, 12). In humans, continued elevation of non-esterified fatty acids (NEFAs) results in decreased secretion of INS and dysfunction of pancreatic β cells, which in turn, leads to the development of DM: a phenomenon termed as lipotoxicity (13, 14).

One of many major consequences of obesity in humans is metabolic syndrome (MS). MS is defined as the set of risk factors associated with cardiovascular disease, one of the most serious lifestyle-related diseases in humans. These metabolic risk factors have extracted enormous burden on national health-care systems and budgets in an increasing numbers of countries (15) and have been the major focus of international research. During the late 1980s, many studies focused on such collection of symptoms in order to define lifestyle diseases. Syndrome X, defined by Reaven (16), included INS resistance, hypertension, GLU intolerance, and hypertriglyceridemia. “The deadly quartet,” described by Kaplan (17), included GLU intolerance, hypertriglyceridemia, hypertension, and upper-body obesity. DeFronzo and Ferannini (18) recognized that INS resistance was commonly associated with such conditions and proposed the term “insulin resistance syndrome.” Then, Matsuzawa et al. (19) proposed the pathophysiological importance of visceral fat deposition in the development of metabolic dysfunctions and introduced the term “visceral fat syndrome.” The above four pathophysiological concepts shared similar conditions such as INS resistance, GLU intolerance, hypertension, hypertriglyceridemia, and obesity, and later referred to as the “metabolic syndrome.” In 2006, International Diabetes Federation (IDF) published a consensus guideline (20), describing a list of diagnostic criteria for MS (Table 1). The diagnostic criteria included waist circumference (WC), TG, cholesterol, GLU concentrations, blood pressure, and the reference values for each criterion. In humans, each component of MS is an independent risk factor for arteriosclerosis, chronic kidney disease, and non-alcoholic steatohepatitis (21). A set of MS diagnostic criteria is a useful tool in preventative medicine as it alerts for an implementation of early environmental, medical, and lifestyle interventions.

**Table 1:** **Human metabolic syndrome diagnostic criteria (20)**.

(1) WC (cm)	≥90 (M); 80 (F) (Japanese)
(2) Triglyceride (mg/dL)	>150
(3) HDL-C (mg/dL)	<40 (M), 50 (F)
(4) Blood pressure (mmHg)	≥130/85
(5) FPG (mg/dL)	≥100 or with T2DM

*WC, waist circumference; HDL-C, HDL-cholesterol; FPG, fasting plasma glucose; M, male; F, female; T2DM, type 2 diabetes mellitus*.

As in humans, obesity-associated diseases and metabolic abnormalities are common in dogs and cats (22, 23). Similar pathophysiological path and disease progression to the development of MS are expected in companion animals. In cats, obesity is associated with the development of INS resistance (24) and type 2 diabetes mellitus (25–28) and is considered a good human MS model (29). Whereas in dogs, symptoms such as obesity, hyperglycemia, and hyperlipidemia are commonly seen but are not considered to directly precede the development of INS resistance and DM (30) In fact, over 50% of DM cases in dogs are type 1, whereas type 2 is much more common in feline (80–95% DM cases) (31). Therefore, the use of the term “metabolic syndrome” in dogs can be considered a misconception (30, 32).

Such species-difference is due to the unique characteristics of feline GLU and lipid metabolism (33). Cats lack one of the rate-limiting enzymes that mediate glycolysis in the liver (34). Hexokinases mediate the conversion of GLU to GLU-6-phosphate as the first step of glycolysis. Hexokinases I–III are present in cats as in dogs and are most active in low blood GLU concentration. Unlike in dogs, lack of hexokinase IV (glucokinase) in cats causes inability to process blood GLU at high concentration and a subsequent accumulation of excess energy as TG in adipose tissues. Additionally, the expression levels of mRNA associated with INS signaling pathway, such as INS receptor substrate-1, INS receptor substrate-2, phosphatidylinositol 3-kinase P-85 alpha in classical INS-responsive tissues, were significantly lower in cats when compared to those in dogs (35). Furthermore, ADN, an adipokine that improves INS sensitivity, was also lower in cats at normal state (35) and with weight gain (36). Unlike in dogs that maintain circulating ADN even with weight gain (36), ADN seems to play more important role in the development of obesity-related metabolic disturbances in cats. Together, these evidences suggest that cats inherently have lower ability to process GLU, are predisposed to obesity and INS resistance, and to visceral obesity-induced lipid metabolism abnormality just as in humans.

In humans, early detection and intervention of weight gain is crucial under the management of preemptive and preventive medicine. It is advised that weight status and health status of at-risk individuals be closely monitored using quantitative parameters such as body mass index (BMI) (37, 38), blood pressure, cholesterol and GLU levels, and WC size as instructed by IDF guidelines (20). Many publications and organizations offer helpful guidelines to promote healthy lifestyle changes including healthy eating, moderate exercise, and stress reduction (39–42). However, in veterinary medicine, there is no consensus on objective biochemical and mechanical parameters and their reference values to classify the weight status. A commonly accepted evaluation method of weight status is Body Condition Score (BCS) (43, 44), which is a semi-quantitative assessment method employing subjective visual observation and palpation of an observer. Such evaluation method is a useful initial screening tool, but subject to interobserver variations and underestimation of the body fat level. Furthermore, it does not reflect the true metabolic and health status of a companion animal because it does not directly measure adipose status. Many evidences suggest the significance of adipose status, such as adipose depot distribution (subcutaneous vs visceral) and adipocyte size (small vs large) in adipokine synthesis and metabolic health (8, 45). In humans, anthropometric measurements such as BMI and WC are considered to correlate well with adipose status (46, 47), but such measurements are not yet available in veterinary medicine. Quantitative, objective measurement methods of body composition in companion animals have been researched in scientific studies. They include ultrasound imaging, computed tomography (CT), magnetic resonance imaging, dual-energy X-ray absorptiometry (DEXA), bioelectrical impedance analysis (BIA), and deuterium oxide dilution method (48, 49) They show good reliability in determining body composition in dogs and cats, but highly inaccessible in veterinary practices. Additionally, most of them measure total body fat and do not quantitatively distinguish between subcutaneous adipose tissue (SAT) and visceral adipose tissue (VAT). Previously, many researchers have introduced various quantitative parameters such as lipid concentrations (50, 51), lipoprotein profiles (52, 53) oxidized low-density lipoprotein (54), and their reference values to evaluate the quality of weight gain.

Practical methods of overweight/obesity diagnosis and classification, employing such measurable biochemical parameters, which reliably reflect adipose status, true metabolic and health status in cats, are in need.

The aim of this manuscript is twofold. First, it presents our preliminary feline obesity study evaluating biochemical and mechanical changes associated with weight gain. In this study, we observed the effects of weight gain on energy metabolism using metabolome markers, such as ADN and proinflammatory cytokines in correlation with other common biochemical parameters in 14 clinically healthy cats of varying BCS. Further, we evaluated the visceral fat accumulation *via* CT imaging and laparoscopic examination and assessed adipocyte type and size histologically in three cats of varying BCS. Second, it proposes the novel diagnostic and classification method of feline obesity and MS, based on the established diagnostic criteria of human MS and our preliminary study data. A set of objective, quantitative biomarkers necessary for early diagnosis and classification of feline obesity is introduced. By defining stage, degree, and quality of weight gain, a guide for interventions (exercise, therapeutic diet, and medications) appropriate for each stage of weight status can be established. Together, they present a feline-specific classification of obesity and MS.

## Animals and Methods

### Animals

Sixteen client-owned cats (0–16 years old; female: *n* = 5; male: *n* = 11) of varying BCS from 6 veterinary hospitals in Kanto area were entered into the study. After medical evaluation, clinically healthy 14 subjects were assigned to 4 groups based on the 5-point scale BCS system (31, 32) described as follows: BCS 1, very thin; BCS 2, underweight; BCS 3, ideal; BCS 4, overweight; and BCS 5, obese. Seven subjects fell into the BCS 3 category, five subjects in BCS 4, and two subjects were in BCS 5. Ethical approval was obtained from the Nippon Veterinary and Life Science University Animal Research Committee. Informed consent was obtained from each client.

### Plasma Analysis

Pre-prandial blood was collected from jugular vein into heparinized plastic tubes. These samples were immediately centrifuged at 1,700 × *g* for 10 min at 4°C to obtain plasma, and the samples were stored at −80°C until use. Plasma GLU, TG, total cholesterol (TC) concentrations and plasma aspartate aminotransferase (AST), alanine aminotransferase (ALT) activities were measured using autoanalyzer (JCA-BM2250, JEOL Ltd., Tokyo, Japan) with the manufacturer’s reagents at Monolis Inc., Tokyo, Japan. Plasma non-esterified fatty acid (NEFA) concentrations were measured with a commercial kit (NEFA-C test, Wako Pure Chemical Industries, Ltd., Tokyo, Japan). Plasma INS and ADN concentrations were measured with a commercial kit (Cat Insulin ELISA kit, Shibatagi Co., Ltd., Gunma, Japan; Mouse/Rat Adiponectin ELISA kit, Otsuka Pharmaceutical Co., Ltd., Tokyo, Japan), respectively. Statistical analysis for plasma parameters was performed by Mann–Whitney *U*-test (*p* < 0.01).

### Laparoscopic Evaluation

Laparoscopic abdominal evaluations of three representative feline subjects (one each from BCS 3, 4, and 5 groups) were performed. Each subject was premedicated with intravenous injections of atropine sulfate at a dose of 0.025 mg/kg, butorphanol at 0.1 mg/kg, diazepam at 0.3 mg/kg, and cefazolin at 20 mg/kg. Anesthetic induction was done using thiopental sodium injection (12.5 mg/kg) and maintained with 2% end-tidal concentration of isoflurane administered *via* endotracheal intubation. Intraoperatively, each subject was administered intravenous fluids at a maintenance dose.

Laparoscopic procedure was performed as described previously (55) using rigid telescope 30° (KARL STORZ Endoscopy Japan, Tokyo), 5 mm trocar–cannulas (Olympus Medical systems, Tokyo), light sources (Xenon nova 175, KARL STORZ Endoscopy Japan, Tokyo), automatic carbon dioxide (CO_2_) insufflator (Electronic Endoflator, KARL STORZ Endoscopy Japan, Tokyo), and video imaging systems (LMD-2450 MD, Sony Corporation, Tokyo). A two-port entry ventral midline approach was performed with a telescope placed through the first 5-mm-cannula at 1 cm caudal to the umbilicus. The second 3-mm cannula was placed at 2–3 cm lateral to the midline on either side. With the use of 5-mm cup biopsy forceps, the edge of a liver lobe was gently grabbed, twisted, and retracted toward the instrument port. Care was taken to avoid rough handling that may lead to excessive hemorrhage and tearing. The same procedure was repeated for intraabominal fat (omental fat). Subcutaneous fat was collected at the time of trocar insertion at the site of abdominal incision.

### CT Imaging

The same three subjects used for laparoscopic evaluation were maintained under anesthesia as described above for CT imaging of the abdomen. All CT images were obtained using a multi-slice CT (BrightSpeed 8 rows, GE Healthcare, Tokyo, Japan). Imaging conditions were as follows: a tube voltage of 120 kV, tube current of 118 mA, and imaging slice thickness of 1.25 mm. Scanned images were reconstructed by RETRO recon reconstruction function of the CT scanner, then processed using a built-in scanner.

### Biopsy Tissues

Parts of liver, omental, and subcutaneous fat were fixed with 10% neutral-buffered formalin and embedded in paraffin wax. Cut sections (4 µm) were stained with hematoxylin and eosin (HE) for histological examination. Formalin-fixed tissues were also frozen, sectioned at 10-µm thickness, and stained with oil red O. Specimen preparation and histopathological evaluation were performed by one clinical pathologist.

## Results

Table 2 shows the results of plasma biochemical measurements. Mean concentration of ADN in BCS 3 cats was significantly higher than those in BCS 4. ADN concentration of 1 subject in BCS 5 showed over 16-fold and 4-fold decrease compared to those in BCS 3 and BCS 4 groups, respectively. Conversely, TNFα concentration of BCS 5 (1 subject) was over 11-times and 9-times higher than those in BCS 3 and BCS 4, respectively. Similarly, TG and NEFA concentrations showed increasing tendency with increasing weight status.

**Table 2:** **Comparison of plasma parameters between cats of Body Condition Score (BCS) 3, 4, and 5 groups**.

	BCS 3 (7)	BCS 4 (5)	BCS 5 (2)
Glucose (mg/dL)	102.9 ± 6.7	114.0 ± 16.4	97
Insulin (ng/mL)	1.3 ± 0.1	1.3 ± 0.1	1.8
Triglyceride (mg/dL)	30.0 ± 10.3	48.8 ± 9.0	121.5
Total cholesterol (mg/dL)	149.9 ± 41.6	166.2 ± 12.0	138.0
NEFA (mEq/L)	0.270 ± 0.003	0.295 ± 0.051	0.327
AST (IU/L)	27.4 ± 2.5	29.2 ± 2.1	20
ALT (IU/L)	42.1 ± 4.6	46.8 ± 5.0	54.0
Adiponectin (μg/mL)	10.1 ± 1.6	2.5 ± 0.7*	0.6 (1)
Tumor necrosis factor α (pg/mL)	117 ± 41 (6)	140 ± 38 (4)	1,346 (1)

*Data of BCS 3 and 4 are presented as mean ± SE*.

*Data of BCS 5 are presented as mean*.

*The numbers in parentheses indicate the number of animals examined*.

*Significantly different from the values BCS 3 by Mann–Whitney U-test, p < 0.01.

**Figure 1:**
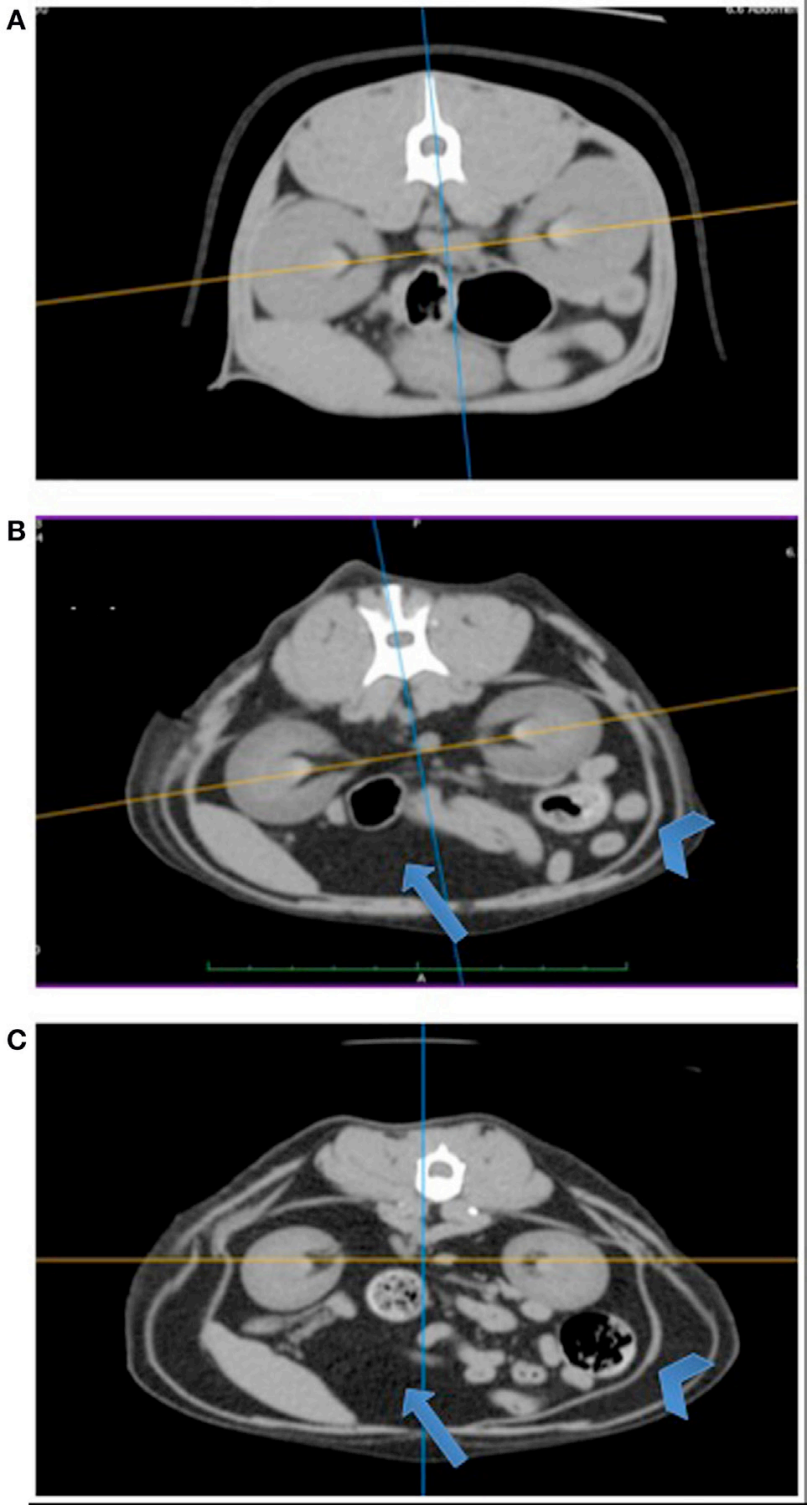
**Comparison of cross-sectional abdominal computed tomography (CT) images at the level of renal pelvis among BCS 3, 4, and 5 cats**. Cross-sectional abdominal CT images at the level of renal pelvis are presented: **(A)** BCS 3 cat; **(B)** BCS 4 cat; and **(C)** BCS 5 cat. Arrows point to intra-abdominal area (visceral fat), and arrowheads point to subcutaneous area. Note with increasing BCS, visceral fat (appeared as black area, arrow) volume increases, occupying more spaces relative to abdominal organs. Subcutaneous fat depot (arrow head) also increases with increasing BCS.

Figure 1 shows three representative cross-sectional CT images (BCS 3, 4, and 5) at the level of renal pelvis. Fat mass, appearing as black image (blue arrow), is visibly thicker in overweight (BCS 4) and obese (BCS 5) cats than in ideal cat (BCS 3), occupying more space relative to the abdominal organs. Furthermore, subcutaneous fat represented by arrow head is visibly thicker in BCS 5 than in BCS 4, and in BCS 4 than in BCS 3.

**Figure 2:**
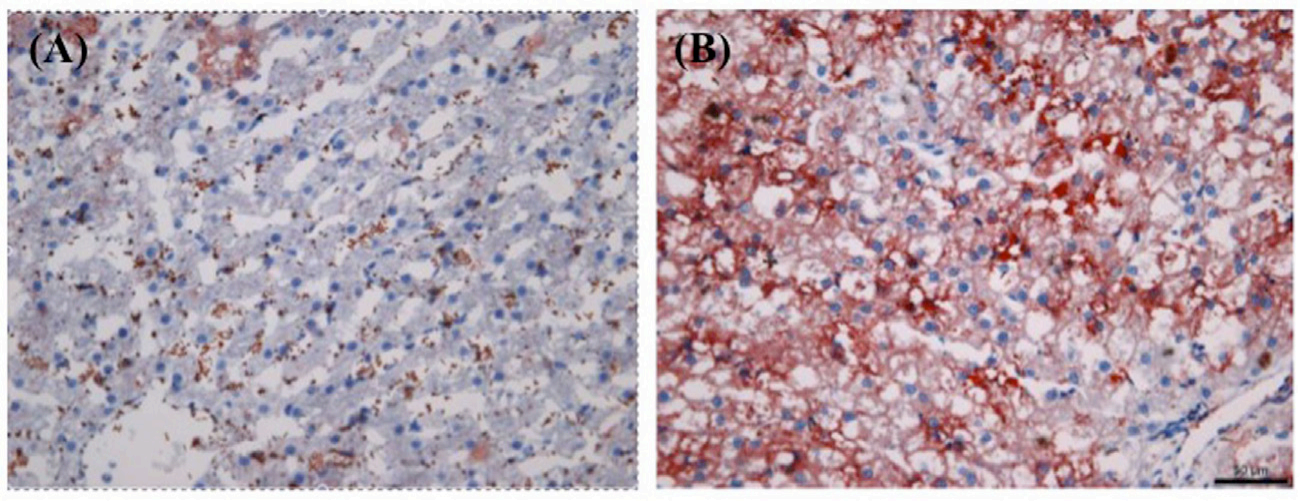
**Histological comparison of biopsied liver tissues between normal [Body Condition Score (BCS) 3] and obese (BCS 5) cats (oil o red stained): (A) BCS 3 (B) BCS 5**. Note the increased level of lipid droplets (stained in red) in BCS 5 liver tissue.

Figure 2 shows the side-by-side comparison of histological images of liver as follows: (A) normal cat liver, oil red O stained; (B) obese cat liver, oil red O stained. As evident, there is no significant finding noted in normal cat liver sample. In obese cat liver, there is an increase in small lipid droplets (shown in orange color, oil o red) at lobular margin. Final histopathological diagnosis was concluded as “lipid droplet accumulation in lobular marginal hepatocytes with no signs of hepatic lipidosis” in obese cat (BCS 5). The liver was accumulating lipids but was not showing pathology as evidenced by the normal activity levels of ALT and AST.

**Figure 3:**
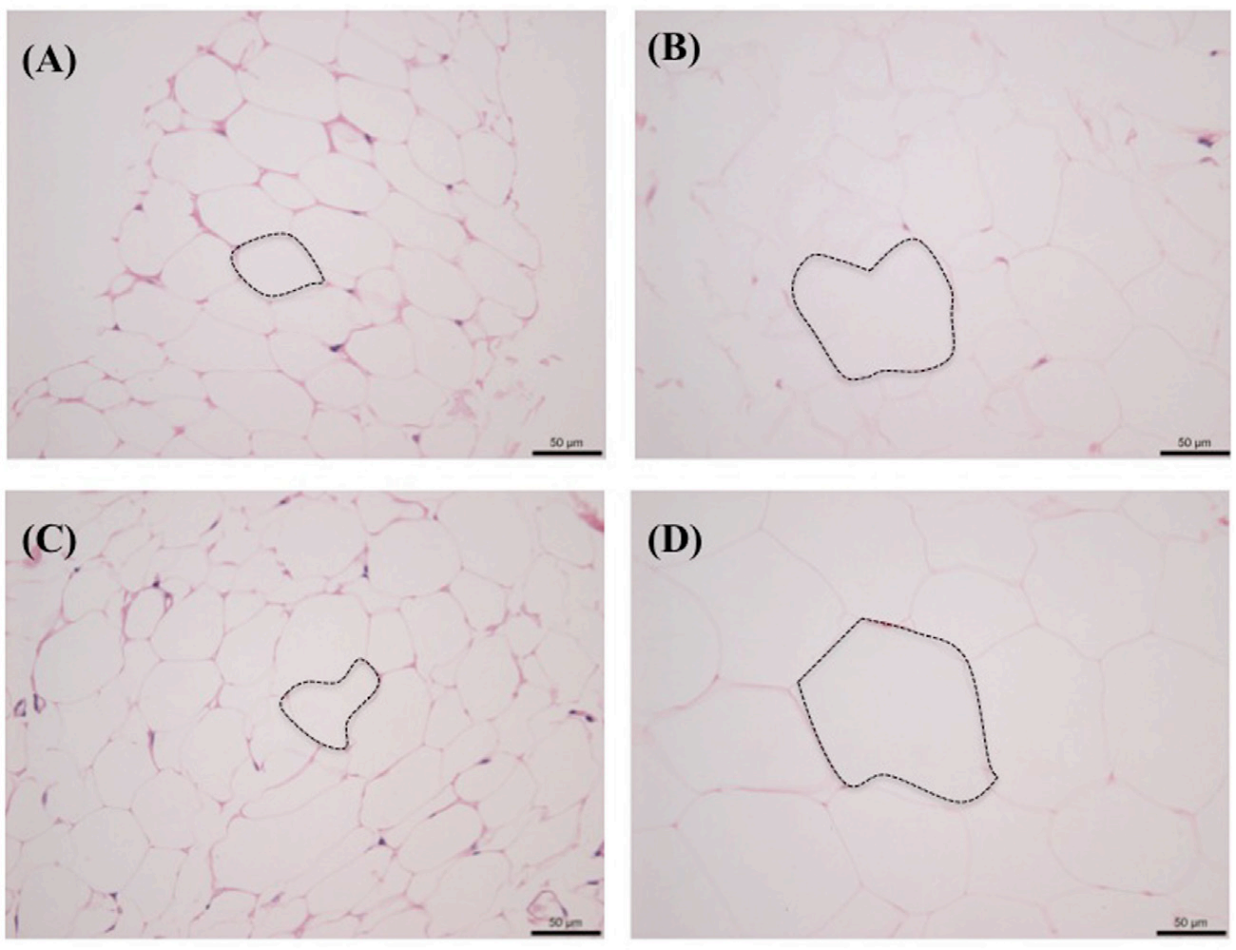
**Histological comparison of subcutaneous and omental fat between normal [Body Condition Score (BCS) 3] and obese (BCS 5) cats: (A) omental fat, BCS 3; (B) ometal fat, BCS 5; (C) subcutaneous fat, BCS 3; and (D) subcutaneous fat, BCS 5**. Note the increased adipocyte size (emphasized with dotted lines) and decreased number of adipocytes with increased BCS.

Figure 3 shows the side-by-side comparisons of HE-stained histological images of subcutaneous and intra-abdominal (omental) fat as follows: (A) normal cat subcutaneous fat, (B) obese cat subcutaneous fat, (C) normal cat omental fat, and (D) obese cat omental fat. Regardless of fat location, there were visibly less adipocytes per field in obese cat samples and larger size adipocytes compared to those in normal cat samples.

## Discussion

In our preliminary feline study, changes in adipose depot distribution and adipocyte sizes in correlation with changes in BCS and metabolome markers were evaluated.

The results showed that with increasing BCS, body fat distribution and adipocyte size shifted from subcutaneous to visceral, and small to large size, respectively. Previous studies suggest the significance of adipose depot distribution (subcutaneous vs visceral) and adipocyte size (small vs large) and age (immature vs mature) (56) in ADN synthesis and secretion (8, 41).

Evidently, our overweight/obese subjects showed lower circulating ADN concentration and more TNFα concentration, showing the mutually antagonizing relationship of ADN and TNFα in concordance with the previous studies (57–59). The decline in ADN concentration, and increase in adipocyte hypertrophy and VAT appeared earlier than changes in liver tissues and common biochemical predictors, such as GLU, liver values, and lipid markers, were evident. Decline in ADN, “beneficial adipokine,” and elevation in TNFα, “harmful adipokine,” in overweight and obese cats, with visceral fat accumulation and adipocyte hypertrophy, support the positive correlation between visceral obesity, metabolic disturbances, and degree of pathology in feline obesity.

**Figure 4:**
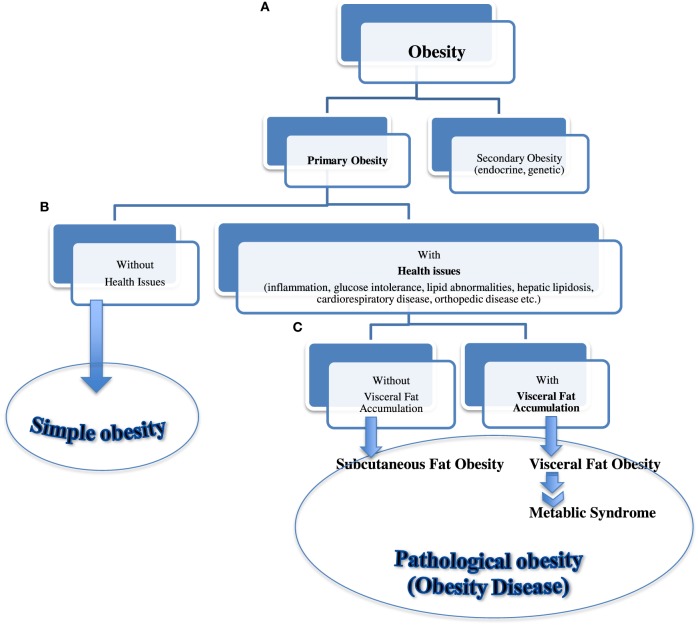
**Classification of feline obesity**. A flow chart showing classification of feline obesity as a guide for early diagnosis and intervention. Obesity is classified by **(A)** its cause, **(B)** presence of associated disease, and **(C)** location of fat depot.

Based on our preliminary study data and previously established human diagnostic criteria (Table 1), we further propose a new “Feline metabolic syndrome diagnostic criteria” (Table 3) and a novel “classification of feline obesity” (Figure 4). Note that central (abdominal) obesity, which is easily assessed by WC in humans, is replaced by BCS. Furthermore, we added ADN as the last criterion as an indicator of visceral fat volume (central obesity) and metabolic health. Unlike in humans, evaluation of weight status using height (m) and weight (kg), as described by BMI, is not practical in cats due to the breed and conformation variations. Instead, 5 or 9 scale BCS (43, 44) is used. In our new classification (Figure 4), “secondary obesity” denotes weight gain induced by endocrine or genetic causes. It requires medical management of underlying diseases and is beyond the scope of this manuscript. “Primary obesity” is of our interest and is further divided into “simple” and “pathological” obesity (obesity disease) based on the presence of obesity-associated symptoms. “Obesity disease” is then divided into “visceral fat obesity” and “subcutaneous fat obesity” based on the location of adipose tissue depot. With progressive and excessive accumulation of VAT, cats are at a higher risk of developing MS. In humans, WC is considered a strong predictor of intra-abdominal fat (visceral fat). In cats, ADN concentration is suggested as the biomarker for visceral fat accumulation associated with MS.

**Table 3:** **Feline metabolic syndrome diagnostic criteria**.

(1) Body Condition Score	>3.5/5 or 5/9
(2) Fasting glucose (mg/dL)	>120
(3) Triglyceride (mg/dL) or total cholesterol (mg/dL)	>165 or >180
(4) Adiponectin (μg/mL)	<3.0

Adiponectin is a cytokine secreted by adipocytes. It is involved in GLU and lipid metabolism and exerts direct effects on vasculatures, thus positively influences the metabolic health of individuals (27). It modulates the expression of fatty acid transport proteins in liver and skeletal muscle and stimulates fatty acid oxidation and GLU utilization *via* activation of AMP-activated protein kinase (AMPK) (60). AMPK is a sensor of cellular energy status and mediates the effects of adipokines in food intake, body weight, and GLU and lipid metabolism (61). It has protective effects against obesity and INS resistance (61, 62). Additionally, AMPK activation suppresses oxidative stress and inflammatory processes, which are the characteristics commonly seen in obese individuals. ADN also exerts anti-inflammatory actions *via* inhibition of macrophage activity and C-reactive protein, TNFα production, and action. It is considered the “beneficial” adipokine, and its decline in plasma is associated with disease processes, such as obesity, INS resistant type 2 diabetes, and coronary artery disease in humans (7, 27). Unlike other adiopokines, secretion and circulating levels of ADN are inversely proportional to body fat mass (63). Low level of circulating ADN is associated with obesity and MS (5). Generally, it is considered to circulate at levels inversely proportional to body fat content. In fact, ADN concentration increases with an increase in SAT, which generally consists of small adipocytes (64) but decreases with increased visceral fat accumulation (8, 45). Therefore, low circulating ADN is considered a strong predictor of visceral fat obesity, associated with GLU and lipid metabolism abnormalities, and can be used as the diagnostic tool for MS.

## Conclusion

As for many other non-communicable diseases, the optimal management of MS is prevention. Successful identification of risk factors and classification of feline obesity can be useful guides when establishing early intervention and management. Our study results support our novel “classification of feline obesity” (Figure 4) and “Feline metabolic syndrome diagnostic criteria” (Table 3), suggesting the need to complement ADN measurement with VAT volume to better understand the pathogenesis of metabolic disturbances in the feline population. Changes in ADN and VAT seemed to reflect the pathophysiological derangements leading to MS much earlier than the common biochemical predictors such as GLU, liver values, NEFA, TG, and TC.

This was a preliminary study to validate our novel classification and diagnostic criteria of feline obesity and MS. Limitations of our study include small number of samples and biological and environmental variables (age, genetics, diet, and exercise), which are inevitable when client-owned animals are utilized. Also, there is no established reference value for feline ADN, thus we used comparison of values among individuals from various BCS. Furthermore, in this study, changes in amounts of SAT and VAT, adipocyte size, and numbers were only visually evaluated. More refined quantitative studies employing direct measurement of adipocytes (45), BIA (65), DEXA (66), or special calculations to measure SAT and VAT volumes (67) on controlled subjects should be performed in the future. In human medicine, a combination of CT scanning and body fat analysis software is often utilized to measure body fat in MS management. No such software specialized for body fat measurement has been developed for veterinary subjects. However, the application of human body fat analysis software has been shown to be useful in a study conducted by Kobayashi et al. (67). Such further studies will help establish reference values for feline ADN concentration and VAT volume that reflect the degree of pathology.

Metabolic syndrome represents a cluster of medical conditions and is considered an optimal target of preemptive medicine. In preemptive medicine, multifaceted factors such as genomics, metabolomics, and environmental associations need to be elucidated. Establishment of diagnostic criteria based on such data will facilitate early diagnosis and interventions and may serve as an established model in the field of veterinary preemptive medicine.

## Author Contributions

YO contributed to the conception and design of the work and drafted the work. MK and MS contributed to the acquisition, analysis, and interpretation of data. TA contributed to the design of the work and final approval of the version to be publication. All authors read and approved the final work.

## Conflict of Interest Statement

The authors declare that the research was conducted in the absence of any commercial or financial relationships that could be construed as a potential conflict of interest.

## Abbreviations

ADN, adiponectin; AMPK, AMP-activated protein kinase; ALT, alanine aminotransferase; AST, aspartate aminotransferase; BIA, bioelectrical impedance analysis; BCS, Body Condition Score; CT, computed tomography; DEXA, c; DM, diabetes mellitus; GLU, glucose; IDF, International Diabetes Federation; INS, insulin; MRI, magnetic resonance imaging; MS, metabolic syndrome; NEFA, non-esterified fatty acid; TC, total cholesterol; TG, triglyceride; TNFα, tumor necrosis factor α; T2DM, type 2 diabetes mellitus; SAT, subcutaneous adipose tissue; VAT, visceral adipose tissue; WC, waist circumference.
